# Input data for inferring species distributions in Kyphosidae world-wide

**DOI:** 10.1016/j.dib.2016.06.043

**Published:** 2016-07-05

**Authors:** Steen Wilhelm Knudsen, Kendall D. Clements

**Affiliations:** School of Biological Sciences, University of Auckland, Auckland, New Zealand

**Keywords:** Sea chub, Drummer, Kyphosus, Scorpis, Girella

## Abstract

Input data files for inferring the relationship among the family Kyphosidae, as presented in (Knudsen and Clements, 2016) [1], is here provided together with resulting topologies, to allow the reader to explore the topologies in detail. The input data files comprise seven nexus-files with sequence alignments of mtDNA and nDNA markers for performing Bayesian analysis. A matrix of recoded character states inferred from the morphology examined in museum specimens representing Dichistiidae, Girellidae, Kyphosidae, Microcanthidae and Scorpididae, is also provided, and can be used for performing a parsimonious analysis to infer the relationship among these perciform families. The nucleotide input data files comprise both multiple and single representatives of the various species to allow for inference of the relationship among the species in Kyphosidae and between the families closely related to Kyphosidae. The ‘.xml’-files with various constrained relationships among the families potentially closely related to Kyphosidae are also provided to allow the reader to rerun and explore the results from the stepping-stone analysis. The resulting topologies are supplied in newick-file formats together with input data files for Bayesian analysis, together with ‘.xml’-files. Re-running the input data files in the appropriate software, will enable the reader to examine log-files and tree-files themselves.

**Specifications Table**

**Table:** 

Subject area	Biology, Genetics and Genomics
More specific subject area	Phylogenetics and Phylogenomics
Type of data	*Nexus file*,.*xml file, newick tree files*, *Nexus-input file*, *tree-file in newick and nexus file-formats for all phylogenetic trees inferred*
How data was acquired	*Sequence data was obtained by PCR using various primers listed by*[1], [2], [3], [4], [5], [6], [7], [8]*and Sanger sequencing as described by*[1]*and aligned using the MAFFT*[9]*plugin in the software Geneious v. R*7 [10]. *Alignments were exported as nexus-files (dataset A*1–*A*7*). The software packages MrBayes v*.3.2 [11]*and BEAST v*.1.8.0 [12]*was used to analyze the resulting alignments. With resulting logfiles examined in Tracer v*.1.5 [13]*, and topologies visualized using FigTree* 1.4.2 [14]*. The software PAUP*[15]*was used to analyze the morphological nexus-input file*
Data format	*Analyzed*
Experimental factors	*A detailed description of steps and settings for each analysis can be found in the material and methods section in the study by*[1]. *All settings used in MrBayes and BEAST is provided in input-nexus-files and BEAUti input-file and in the supplementary tables included in*[1]
Experimental features	*Tissue samples were obtained from vouchered specimens of Kyphosidae and other perciform species. Extractions of DNA was performed on* 109 *tissue samples, and used for PCR amplification and Sanger Sequencing*. *Visual inspection of sequence reads from mitochondrial DNA and nuclear DNA made it possible to assemble alignments that subsequently could be used for preparing nexus-input files for analysis in MrBayes and BEAST to infer the evolutionary relationship among Kyphosidae and closely related perciform species*. *Best partitioning and nucleotide substitution models and nucleotide substitution saturation was inferred using PartitionFinder*[16]*and DAMBE*[17], *respectively*. *Vouchered specimens of fishes, listed by*[1], [19]*together with examination of* 584 *museum specimens made it possible to evaluate character states for* 152 *morphological characters for* 43 *species*. *Character ranges were divided in to a maximum of ten bins, and median character state values were assigned for each polymorphic character*. *The resulting morphological data matrix was subsequently analyzed in PAUP*
Data source location	*n/a*
Data accessibility	*Data is within this article*

**Value of the data**

• The provided nexus-files holds sequence data and alignments and can be directly utilised in future studies in the evolution of perciform fishes.
• The phylogenetic trees supplied in newick-file format together with tables presented by [1] show that samples of *Kyphosus* and *Neoscorpis* collected worldwide groups in clades reflecting the presence of 12 species of Kyphosidae in total. This will facilitate species identification by matching mitochondrial nucleotide sequences of future kyphosid catches by allowing for comparison with sequences obtained in this study.
• Supplied tree-files in newick format, and the evolutionary relationship inferred by [1] supports Scorpididae as the closest related family to Kyphosidae, and these files allow the reader to see the other closely related families to Kyphosidae by using a phylogeny visualisation tool such as FigTree v.1.4.2 [14].

## 1 Data

Alignments of nucleotide nuclear and mitochondrial data are provided as ‘.nex’-files and ‘.xml’-files for analysis in MrBayes [11] and BEAST [12], respectively. Phylogenetic trees derived from the analysis are supplied in ‘.new’-file format. All tissue samples, vouchered specimens, sampling date and locality can be found in [1]. A matrix of 152 recoded morphological characters is provided as a ‘.nex’-file [morph_pars_matrix.nex] that comprises 43 taxa representing the following families: Dichistiidae, Girellidae, Kyphosidae, Microcanthidae and Scorpididae according to the treatment of these groups as families as presented by [18]. This morphological data matrix file can be opened in Mesquite v.3.04 [19].

## 2 Experimental design, materials and methods

Detailed descriptions of how DNA sequences were obtained and analysed can be found in the material and methods section in [1], which provides detailed information on samples, PCR set-ups, reagents and sequencing and subsequent analysis of nucleotide data. The supplementary material provided in [1] also lists specific primers used for amplification of DNA, and lists the National Centre for Biotechnology Information (NCBI) GenBank accession numbers for all included sequences. The sequence data obtained by [1], were used to prepare the input data-files supplied here with this data-article, and were then used to infer the relationship among Kyphosidae, *sensu* [18], and other closely related families. The data used for inferring the relationship in Kyphosidae [1] comprises seven nexus-files [mb_A1_SFig02_nmt.nex; mb_A2_SFig01_mt.nex; mb_A3_SFig03_n.nex; mb_A4_SFig04_r1.nex; mb_A5_SFig05_r2.nex; mb_A6_SFig06_t4.nex; mb_A7_fig02input.nex] with sequence alignments of mtDNA and nDNA markers inferred from 47 and 109 tissue samples. A BEAuti-file [BEAST_A7_03_fig03.beautiv180] for creating the xml-file [beast_A7_03_fig03.xml] that is used as input for a BEAST analysis is also supplied, this xml-file is based on dataset A7 using the Bayesian inferred tree as a starting tree. An ‘empty’ version of the ‘xml’-file (i.e. without nucleotide data) is also provided [beast_A7_03E_fig03.xml] to allow for a check of the prior settings applied in the ‘xml’-file containing nucleotide data. Seven additional ‘.xml’-input files for performing Stepping Stone (ss) analysis in BEAST v.1.8.0 is also supplied. These ‘.xml’-input files [bk86_07_36ss_no_constraints.xml;bk86_07_37ss_kyp_gir.xml;bk86_07_38ss_kyp_sco.xml; bk86_07_39ss_kyp_mic.xml; bk86_07_40ss_kyp_kuh.xml; bk86_07_41ss_kyp_ter.xml; bk86_07_42ss_kyp_opl.xml] has taxa representing the families: Girellidae, Kuhliidae, Kyphosidae, Microcanthidae, Olegnathidae, Scorpididae and Terapontidae constrained into different combinations of monophyletic groups, as described by [1] to allow for ss-test of which family can be considered most closely related to Kyphosidae. The ‘.xml’-input files are only provided with inclusion of nucleotide data, but can be prepared without nucleotide data (i.e. as empty ‘.xml’-files for test of prior performance) from the BEAuti-file [BEAST_A7_03_fig03.beautiv180] used to prepare all other ‘.xml’-files. The resulting marginal-likelihood values obtained from running each of these ss-‘.xml’-files four times in parallel are also included, together with the resulting consensus trees, the ‘_mean_tree.tre’-files, which can be viewed in Mesquite v. 3.04 [19].

A nexus-input file [morph_pars_matrix.nex] with morphological character states inferred for 43 species, representing 584 museum specimens listed by [18] is also supplied. The resulting topologies are also supplied in newick-file formats for all topologies inferred [mb_A1_SFig02_nmt.nex.con.nwk; mb_A2_SFig01_mt.nex.con.nwk; mb_A3_SFig03_n.nex.con.nwk; mb_A4_SFig04_r1.nex.con.nwk; mb_A5_SFig05_r2.nex.con.nwk; mb_A6_SFig06_t4.nex.con.nwk; mb_A7_fig02tree.nwk; beast_A7_03_fig03.nwk; morph_pars_matrix_tree.nwk]. These topologies can be explored by the reader using FigTree v.1.4.2. [14]. The settings applied for the analysis in MrBayes and BEAST is described in the materials and methods section by [1] and is also included in the data input files for MrBayes, BEAST and PAUP supplied with this zipped data file.
